# Epidemiology and antimicrobial resistance trends in *Haemophilus parainfluenzae* in a tertiary care hospital (2018–2022)

**DOI:** 10.1128/spectrum.01039-26

**Published:** 2026-07-15

**Authors:** Lucía Saiz-Escobedo, Irene Cadenas-Jiménez, David Sánchez, Xènia Camprubí-Márquez, Sara Calvo-Silveria, Guillem López de Egea, Dàmaris Berbel, Rosa Maria Costa, Jordi Cámara, Maria Angeles Dominguez-Luzon, Carmen Ardanuy, Arkaitz Imaz, Aida González-Díaz, Sara Marti

**Affiliations:** 1 Microbiology Department, Hospital Universitari de Bellvitge, IDIBELL-UB 16383, Barcelona, Spain; 2 Department of Pathology and Experimental Therapeutics, School of Medicine, University of Barcelona 16724, Barcelona, Spain; 3 Research Network for Respiratory Diseases (CIBERES), ISCIII 568067 , Madrid, Spain; 4 Research Network for Infectious Diseases (CIBERINFEC), ISCIII 637284 , Madrid, Spain; 5 HIV and STI Unit, Infectious Diseases Department, Hospital Universitari de Bellvitge, IDIBELL, University of Barcelona 16383, Barcelona, Spain; 6 Department of Medicine, School of Medicine, University of Barcelona 368901, Barcelona, Spain; MultiCare Health System, Tacoma, Washington, USA

**Keywords:** whole-genome sequencing, mobile genetic elements, integrative and conjugative elements, ESBL, β-lactamase, multidrug resistance, *Haemophilus parainfluenzae*, genomic epidemiology, sexually transmitted infections, capsular operon

## Abstract

*Haemophilus parainfluenzae* is increasingly recognized as a reservoir of antimicrobial resistance in urogenital infections. We conducted a retrospective laboratory-based study including all *H. parainfluenzae* isolates collected at a tertiary hospital in Barcelona between 2018 and 2022. Antimicrobial susceptibility testing was performed for all isolates, and whole-genome sequencing was conducted for multidrug-resistant (MDR) and capsulated strains. A total of 529 isolates from 504 individuals were analyzed (258 respiratory, 223 urogenital, and 48 other sources). Overall, 85/529 (16.1%) isolates were MDR, with a significantly higher prevalence among urogenital compared with respiratory samples (30.5% vs 6.2%). Among symptomatic urogenital episodes caused by MDR isolates, *H. parainfluenzae* was the only sexually transmitted infection (STI)-associated urogenital pathogen organism recovered in 65.3% of cases. Resistance was mediated by acquired genes and target-site substitutions, including β-lactamases (*bla*_TEM-1_, *bla*_ROB-1_, and *bla*_CTX-M-15_), PBP3 substitutions, macrolide efflux determinants, quinolone resistance-determining regions mutations, and determinants conferring resistance to chloramphenicol, folate pathway inhibitors, and tetracyclines. Mobile genetic elements were frequent, including diverse integrative and conjugative elements, chromosomal insertions, and small β-lactamase plasmids distributed across genetically diverse lineages, with no evidence of a dominant MDR clone. Capsular operons were detected in 6.6% of the isolates, mainly urogenital, including two operons not observed previously at our center; 26/35 capsulated isolates were MDR, and capsular loci occurred across multiple lineages. These findings support *H. parainfluenzae* as an STI-associated urogenital pathogen and highlight the widespread distribution of mobile resistance determinants, reinforcing the need for continued genomic surveillance.

**IMPORTANCE:**

*Haemophilus parainfluenzae* is increasingly recognized as a potential pathogen in urogenital infections, particularly in sexually transmitted infection settings, yet its clinical relevance remains underappreciated. In this study, we show that multidrug-resistant strains are frequently associated with urogenital samples and can be isolated as the only detectable pathogen in symptomatic patients. Our findings highlight the important role of mobile genetic elements, including integrative and conjugative elements and small plasmids, in driving the spread of antimicrobial resistance across diverse bacterial lineages. We also identify capsular operons in a subset of isolates, supporting their potential role in niche adaptation and pathogenicity. Together, these results emphasize the need to consider *H. parainfluenzae* in the diagnosis and management of urogenital infections and underscore the importance of continued surveillance to monitor the emergence and dissemination of resistance.

## INTRODUCTION

*Haemophilus parainfluenzae* is a facultative anaerobic gram-negative coccobacillus that forms part of the normal microbiota of the human respiratory and urogenital tracts (1). Although traditionally considered a commensal organism, it is increasingly recognized as an opportunistic pathogen capable of causing a wide range of infections. It has been implicated in respiratory tract infections (2, 3), endocarditis (4), osteomyelitis (5), and other invasive diseases, such as bacteremia (6), septic arthritis (7), and meningitis (8). In recent years, particular attention has been given to its role in urogenital infections, with multiple reports highlighting its association with urethritis (1, 9–11). The increasing detection of *H. parainfluenzae* in urogenital samples suggests that this species may be an under-recognized pathogen in sexually transmitted infections (STIs), emphasizing the need for continued epidemiological surveillance to clarify its clinical relevance.

In parallel, *H. parainfluenzae* has shown a concerning increase in antimicrobial resistance, with the emergence of multidrug-resistant (MDR) and extensively drug-resistant (XDR) strains (12). Resistance has been reported against multiple antibiotic families, including β-lactams, macrolides, fluoroquinolones, chloramphenicol, co-trimoxazole, and tetracycline. This resistance is mainly due to point mutations in key antibiotic target genes or acquisition of resistance determinants by horizontal gene transfer (13). *H. parainfluenzae* has a remarkable ability to acquire foreign genetic material. The presence of mobile genetic elements (MGEs), such as integrative and conjugative elements (ICEs) and transposons (Tn), plays a crucial role in this process, facilitating the exchange of resistance genes between bacterial populations (14, 15).

Resistance to β-lactams in *H. parainfluenzae* is primarily mediated by the production of β-lactamases, such as *bla*_TEM_ and *bla*_ROB_, as well as point mutations in *ftsI*, which encodes penicillin-binding protein 3 (PBP3) (16, 17). Macrolide resistance is mainly associated with the acquisition of the *tet*(M)-MEGA element, which carries *mef*(E) and *msr*(D), encoding efflux pumps that reduce antibiotic efficacy (18, 19). Fluoroquinolone resistance typically arises from mutations in the *gyrA* and *parC* genes, which alter DNA gyrase and topoisomerase IV, decreasing antibiotic binding (20). Chloramphenicol resistance is mediated by chloramphenicol acetyltransferase enzymes, encoded by *cat* genes such as *catA2* and *catS* (13). Resistance to co-trimoxazole is often driven by mutations in *folA*, *folP*, and the *folA* promoter, as well as the acquisition of *sul2* (17, 21). Tetracycline resistance in *H. parainfluenzae* can be attributed to either the acquisition of the *tet*(M)-MEGA element or the presence of an efflux system encoded by *tet*(B)/(C)/(D)/(R) genes. Many of these resistance determinants are disseminated through MGEs (13).

More recently, the detection of the extended-spectrum β-lactamase (ESBL) CTX-M-15 in *H. parainfluenzae* has raised further concerns, particularly as it has been identified in XDR urogenital strains (22). The presence of CTX-M-15 in MGEs indicates a potential for horizontal transfer and, since its initial reporting, additional cases have been described (23, 24), highlighting the importance of genomic surveillance to monitor its dissemination and assess its clinical impact.

In addition to antimicrobial resistance, one virulence factor of interest in *H. parainfluenzae* is the polysaccharide capsule, predominantly identified in urogenital isolates (25). Although its role in this species remains unclear, polysaccharide capsules may contribute to virulence and niche adaptation. The capsular operon (*cap* locus) comprises three different regions. Region I is formed by four common genes (*bexABCD*) that encode the components of the export apparatus, which is responsible for the translocation of the capsular polysaccharides. Region II genes are serotype-specific and are involved in polysaccharide biosynthesis; they comprise three to eight genes. Finally, region III contains two genes (*hcsAB*) encoding proteins involved in polysaccharide transport across the outer membrane (26). Capsular operons in *Haemophilus* spp. have been classified into three groups based on their genetic structure (13). Group 1 includes *H. influenzae* serotypes a and b; group 2 includes *H. influenzae* serotypes c and f, *Haemophilus sputorum* HSPU-type1, and *H. parainfluenzae* HPAR-type1; and group 3 includes *H. influenzae* serotypes d and e, *H. sputorum* HSPU-type2, and *H. parainfluenzae* HPAR-type2. Recently, two new capsular operons have been described in *H. parainfluenzae*: serotype f-like operon (27) and *H. parainfluenzae* HPAR-type4 (28).

Between 2013 and 2017, a previous study conducted at our hospital characterized the epidemiological and genomic profile of *H. parainfluenzae*, highlighting an increasing prevalence of antimicrobial resistance, particularly in urogenital isolates (1, 13). Building on these findings, the present study provides a 5-year follow-up to assess the evolution of resistance patterns, identify the genetic mechanisms underlying MDR phenotypes, and characterize the prevalence and genetic diversity of capsular operons among *H. parainfluenzae* strains.

## RESULTS

### Overview of the collection: epidemiology and resistance

**Fig 1:**
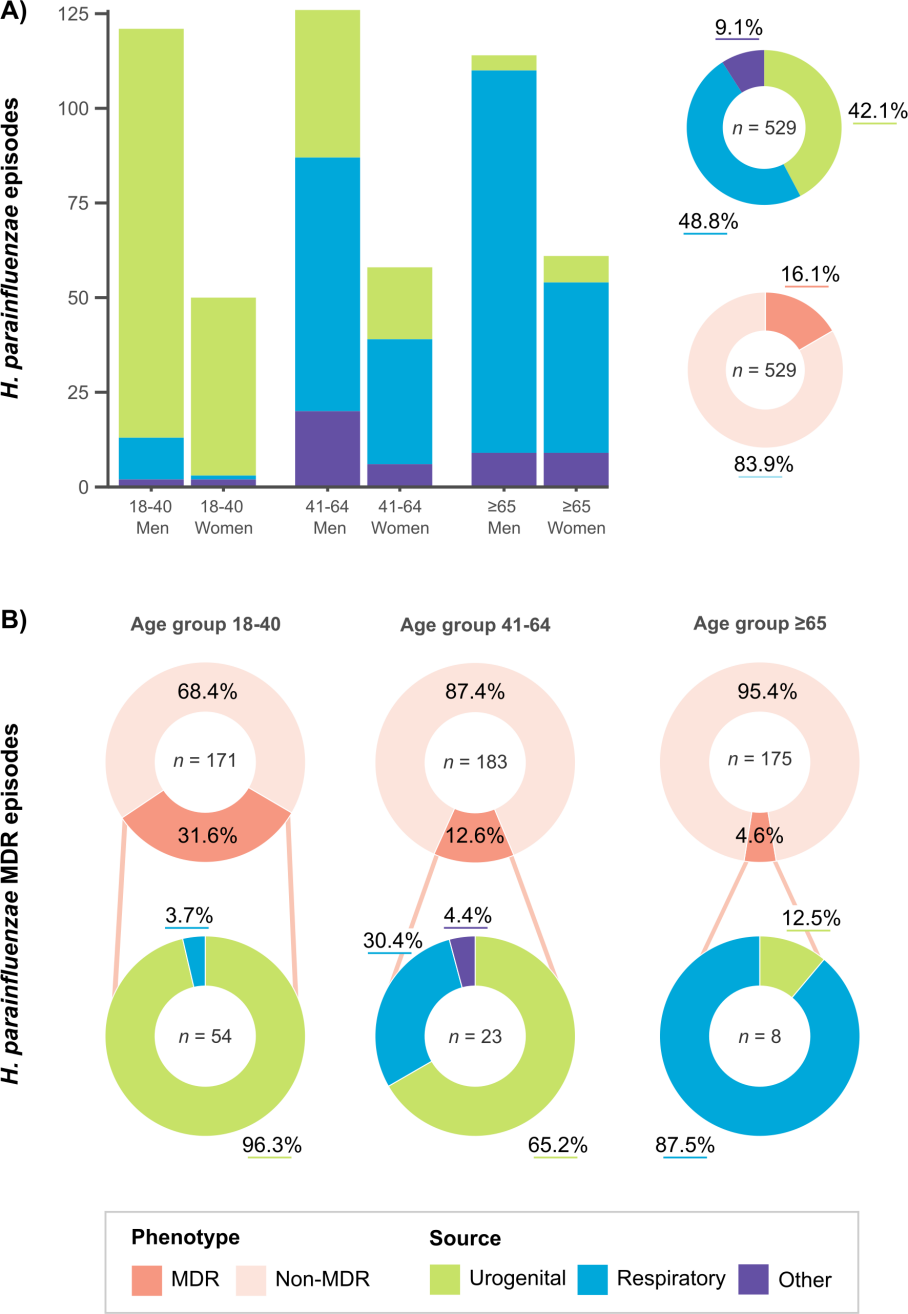
Epidemiology of *H. parainfluenzae* isolates at Hospital Universitari de Bellvitge (2018–2022). (**A**) Distribution of all isolates stratified by sex and age groups: young adults (18–40 years), middle-aged adults (41–64 years), and elderly patients (≥65 years). Bars are subdivided by sample type: urogenital, respiratory, and other samples. Donut charts represent the distribution of sample origin and the global percentage of multidrug-resistant (MDR) isolates. (**B**) Proportion of MDR isolates relative to the total number of isolates within each age group and sample type.

Between 2018 and 2022, a total of 529 *H. parainfluenzae* isolates were obtained from clinical samples of 504 individuals (one isolate per episode). Of these, 258 (48.8%) were collected from respiratory samples, 223 (42.1%) from urogenital samples, and 48 (9.1%) from other sample types (Fig. 1A). Respiratory isolates were mainly obtained from sputum (61.6%) and bronchial aspirates (29.8%), while urogenital isolates were predominantly urethral (49.3%) and vaginal swabs (30.1%). Isolates from other anatomical sites included a heterogeneous group of sample types (Table S1).

Overall, *H. parainfluenzae* was more frequently isolated from male individuals (*n* = 360, 68.1%). The age distribution was relatively balanced across groups (Fig. 1A); however, marked differences emerged when analyzed by sample origin (Table S2A). Urogenital isolates were predominantly recovered from young adults (69.5%), whereas respiratory isolates were mainly obtained from middle-aged (56.6%) and elderly individuals (36.1%). The median age of individuals with urogenital isolates was 32.0 years (IQR 25.0–45.0) (Table S2B), which was significantly lower than that of patients with respiratory isolates (66.5 years [59.0–72.0], *P* < 0.0001). Antimicrobial susceptibility data were available for all 529 *H. parainfluenzae* isolates (Table 1), and resistance was detected across all antibiotic classes, with the highest rates observed for ciprofloxacin, tetracycline, and ampicillin (Table 1). Urogenital isolates showed significantly higher resistance rates than respiratory isolates for most antibiotics, particularly β-lactams, chloramphenicol, cotrimoxazole, and tetracycline, whereas resistance to ciprofloxacin and azithromycin was similarly distributed between sources. Given the clinical relevance of resistant phenotypes, the subset of MDR isolates was analyzed by whole-genome sequencing (WGS).

**TABLE 1:** Resistance rates of *Haemophilus parainfluenzae* in the overall collection and in the MDR subset, by clinical source (2018–2022)^*a*^*^,^*^*e*^

	Overall, *n* (%)	Urogenital, *n* (%)	Respiratory, *n* (%)	Others, *n* (%)	*P* value^*c*^
Antibiotics^*b*^	*n* = 529	*n* = 223	*n* = 258	*n* = 48	
Ampicillin	128 (24.2)	74 (33.2)	49 (19.0)	5 (10.4)	0.01
Amoxicillin/clavulanic acid	46 (8.7)	35 (15.7)	11 (4.3)	0 (0.0)	<0.001
Cefuroxime	51 (9.5)	40 (17.9)	10 (3.9)	0 (0.0)	<0.0001
Cefotaxime	48 (8.9)	39 (17.5)	8 (3.1)	0 (0.0)	<0.0001
Azithromycin	70 (13.2)	39 (17.5)	30 (11.6)	1 (2.1)	NS
Ciprofloxacin	155 (29.3)	77 (34.5)	65 (25.2)	13 (27.1)	NS
Chloramphenicol	49 (9.3)	43 (19.3)	5 (1.9)	1 (2.1)	<0.0001
Cotrimoxazole	112 (21.2)	86 (38.6)	22 (8.5)	4 (8.3)	<0.0001
Tetracycline	134 (25.3)	91 (40.8)	36 (14.0)	7 (14.6)	<0.0001
**MDR**	***n* = 85 (16.1)**	***n* = 68 (30.5)**	***n* = 16 (6.2)**	***n* = 1 (2.1)**	
Ampicillin	76 (89.4)	62 (91.2)	13 (81.3)	1 (100.0)	NS
Amoxicillin/clavulanic acid	42 (49.4)	33 (48.5)	9 (56.3)	0 (0.0)	NS
Cefuroxime	47 (55.3)	38 (55.8)	9 (56.3)	0 (0.0)	NS
Cefotaxime	47 (55.3)	38 (55.8)	9 (56.3)	0 (0.0)	NS
Ceftriaxone^*d*^	43 (50.6)	35 (51.4)	8 (50.0)	0 (0.0)	NS
Meropenem^*d*^	0 (0.0)	0 (0.0)	0 (0.0)	0 (0.0)	NS
Azithromycin	49 (57.6)	37 (54.4)	12 (75.0)	0 (0.0)	NS
Ciprofloxacin	63 (74.1)	51 (75.0)	12 (75.0)	0 (0.0)	NS
Chloramphenicol	44 (51.8)	39 (57.4)	4 (25.0)	1 (100.0)	NS
Cotrimoxazole	75 (88.2)	60 (88.3)	14 (87.5)	1 (100.0)	NS
Tetracycline	84 (98.8)	64 (94.1)	14 (87.5)	1 (100.0)	NS
R to four antibiotic families	35 (41.2)	26 (38.2)	8 (50.0)	1 (100.0)	NS
R to five antibiotic families	19 (22.4)	14 (20.6)	5 (31.3)	0 (0.0)	NS
R to six antibiotic families	19 (22.4)	18 (26.4)	1 (6.3)	0 (0.0)	NS

^*a*^ Percentages were calculated based on the total number of isolates within each clinical source. For the MDR block, percentages were calculated within the MDR subset.

^*b*^ European Committee on Antimicrobial Susceptibility Testing clinical breakpoints for *Haemophilus influenzae* were used.

^*c*^ The *P* values were obtained by comparing urogenital isolates vs respiratory isolates.

^*d*^ Ceftriaxone and meropenem susceptibility were determined only in MDR episodes.

^*e*^ MDR, multidrug-resistant; NS, not statistically significant; R, resistance.

### Prevalence and clinical characterization of MDR cases

Over the study period, 85/529 (16.1%) *H. parainfluenzae* isolates were MDR (Table 1), with a significantly higher prevalence among urogenital isolates (30.5%) compared with respiratory samples (6.2%, *P* < 0.0001), and only sporadic detection in other sources (one isolate).

MDR isolates were significantly more frequent among male patients (69/360, 19.2%) than females (16/169, 9.5%) (*P* < 0.01) and were predominantly recovered from young adults, who accounted for 63.5% of all MDR cases (*P* < 0.0001). Consistent with this pattern (Fig. 1B), MDR strains in young adults were predominantly associated with urogenital samples, whereas MDR isolates from respiratory samples were mainly observed in older age groups.

To further investigate the clinical context of MDR *H. parainfluenzae* infections, we retrospectively reviewed the clinical charts of the affected patients. Only episodes involving urogenital and respiratory isolates were included.

#### Urogenital MDR episodes

Clinical data were available for 64 of the 68 MDR cases, of which 49 were included after excluding asymptomatic cases or those lacking relevant urogenital symptoms. All included episodes presented with symptoms compatible with an STI, with urethritis being the predominant clinical presentation, followed by genital ulcers and vulvovaginitis (Table 2).

**TABLE 2:** Clinical records of symptomatic patients with MDR *H. parainfluenzae* collected from urogenital samples (*n* = 49)^*a*^^,^^*b*^

Characteristics	*n* (%)
Age, median (IQR)	32.8 (25.0–37.0)
Sex (men)	42 (85.7)
Sexual orientation	
No data	9 (18.4)
MSM	23 (54.8)^*c*^
Heterosexual	17 (34.7)
Risk exposures	37 (75.5)
No data	6 (12.2)
Unprotected sex	31 (63.3)
Multiple partners	16 (32.7)
PrEP	6 (12.2)
HIV infection	8 (16.3)
Clinical symptoms	
Urethritis	34 (69.4)
Genital ulcer	7 (14.3)
Vulvovaginitis	6 (12.2)
Pelvic inflammatory disease	1 (2.0)
Balanitis	1 (2.0)
Previous STI	30 (61.2)
Co-infection with other STI pathogens^*d*^	
None (only *Haemophilus parainfluenzae*)^*e*^	32 (65.3)
*Neisseria gonorrhoeae*	6 (12.2)
*Chlamydia trachomatis*	5 (10.2)
*Treponema pallidum*	3 (6.1)
*Gardnerella vaginalis*	2 (4.1)
*Candida albicans*	1 (2.0)
*Trichomonas vaginalis*	1 (2.0)
Antimicrobial therapy	
No data	5 (10.2)
Ceftriaxone IM	2 (4.1)
Ceftriaxone IM and azithromycin OR	14 (28.6)
Ceftriaxone IM and doxycycline OR	4 (8.2)
Ceftriaxone IM, doxycycline OR, and others^*f*^	9 (18.4)
Other regimens^*g*^	15 (30.6)

^*a*^ HIV, human immunodeficiency virus; IM, intramuscular; IQR, interquartile range; MSM, men who have sex with men; OR, oral; PrEP, pre-exposure prophylaxis; STI, sexually transmitted infection.

^*b*^ Inclusion criteria: symptomatic urogenital MDR episodes; asymptomatic cases or records without relevant urogenital symptoms were excluded. Percentages were calculated based on the total number of episodes included.

^*c*^ Percentage was calculated based on the total number of male patients (*n* = 42).

^*d*^ Other STI pathogens detected in culture or PCR in the same clinical episode.

^*e*^ In these 32 cases, *H. parainfluenzae* was the only STI-related pathogen identified by culture. Among them, 23 had negative PCR results for *N. gonorrhoeae* and *C. trachomatis*, and 17 had negative syphilis serology; in the remaining cases, these tests were not requested.

^*f*^ Others include amoxicillin/clavulanic acid OR, azithromycin OR, ciprofloxacin OR, ertapenem IM, and/or penicillin G IM, administered in combination with ceftriaxone IM and doxycycline OR.

^*g*^ Other regimens include a wide range of empirical treatments, such as antifungals (miconazole and fluconazole), clindamycin, fosfomycin, metronidazole, and various oral combinations, including amoxicillin/clavulanic acid, azithromycin, ciprofloxacin, and doxycycline.

Patients were predominantly young adults (median age 32.8 years [IQR 25.0–37.0]) and mostly male, with a high proportion of men who have sex with men. Commonly reported risk factors included unprotected sex, multiple sexual partners, and previous STI diagnoses. Co-infection with other STI pathogens was observed in approximately one-third of the cases, most frequently involving *Neisseria gonorrhoeae* and *Chlamydia trachomatis.*

In 32 cases (65.3%), *H. parainfluenzae* was the only STI-related pathogen isolated in culture. Antimicrobial therapy varied widely due to the absence of specific treatment guidelines for this species. The most frequently administered regimens included intramuscular ceftriaxone combined with oral azithromycin (28.6%) or doxycycline (8.2%). These treatments correspond to therapies prescribed for the clinical episode and were frequently directed towards other concomitant STI pathogens when present. In six cases (12.2%), the initial antimicrobial regimen required adjustment due to persistent symptoms and/or subsequent microbiological findings, requiring the addition of other antibiotics. In the remaining cases, either symptom resolution was documented, or no follow-up information was available.

#### Respiratory MDR episodes

Clinical charts were obtained for all 16 MDR *H. parainfluenzae* respiratory episodes. Three cases were discarded because they were not clearly related to respiratory infection. Therefore, 13 episodes were included in the final analysis. Patients were older (median age 65.7 years [IQR 61.0–69.0]) and predominantly male. Comorbidities and chronic respiratory diseases were common, although detailed clinical characteristics are shown in Table 3. Clinical presentations were heterogeneous, ranging from exacerbations of chronic respiratory conditions, pneumonia, or other acute infections. In all cases, *H. parainfluenzae* was part of a polymicrobial infection, and its pathogenic role could not be unequivocally established. Antimicrobial treatments varied considerably, targeting other suspected pathogens or administered as prophylaxis. Regimens included azithromycin, amoxicillin/clavulanic acid, ceftriaxone, fluoroquinolones, and other broad-spectrum antibiotics.

**TABLE 3:** Clinical records of symptomatic patients with MDR *H. parainfluenzae* collected from respiratory samples (*n* = 13)^*a*^^,^^*b*^

Characteristics	*n* (%)
Age, median (IQR)	65.7 (61.0–69.0)
Sex (men)	8 (61.5)
Underlying conditions	
None	8 (61.5)
Cancer	3 (23.1)
Cardiopathy	4 (30.8)
Chronic liver disease	1 (7.7)
Diabetes	2 (15.4)
Organ transplant or immunosuppression	0 (0.0)
Risk factors	
No data	1 (7.7)
None	7 (53.8)
Alcohol	2 (15.4)
Tobacco exposure	5 (38.7)
Respiratory diseases	
Acute respiratory infection^*c*^	5 (38.5)
Bronchiectasis	3 (23.1)
COPD	3 (23.1)
Pneumonia	2 (15.4)

^*a*^ COPD, chronic obstructive pulmonary disease; IQR, interquartile range.

^*b*^ Inclusion criteria: symptomatic respiratory MDR episodes with supportive clinical evidence of infection; episodes not clearly related to respiratory infection or lacking sufficient documentation were excluded. Percentages were calculated based on the total number of episodes included.

^*c*^ Acute respiratory infection refers to upper or lower respiratory tract symptoms documented during the clinical episode, excluding pneumonia.

### Resistance profiles and genetic determinants in MDR strains

**Fig 2:**
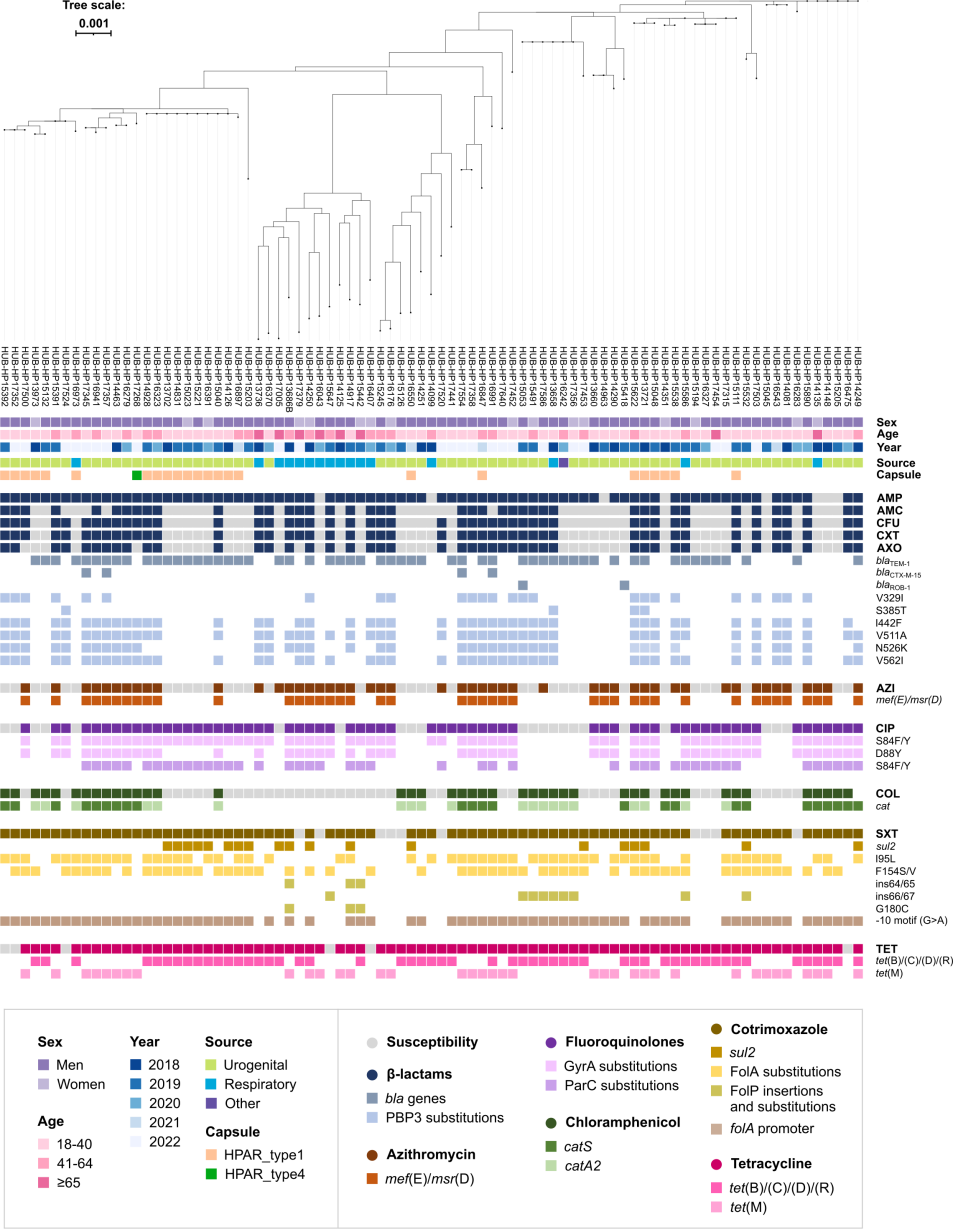
rMLST-based phylogeny and resistance mechanisms of MDR *Haemophilus parainfluenzae* strains. The dendrogram was inferred from pairwise allelic distances across the 53 ribosomal loci (rMLST, PubMLST scheme). Tip labels indicate strain IDs. Colored bars indicate demographic data, biological source, capsule presence, antimicrobial resistance, and the main resistance determinants detected. AMC, amoxicillin/clavulanic acid; AMP, ampicillin; AXO, ceftriaxone; AZI, azithromycin; CFU, cefuroxime; COL, chloramphenicol; CXT, cefotaxime; IP, ciprofloxacin; XT, cotrimoxazole; TET, tetracycline.

The resistance phenotypes observed among MDR *H. parainfluenzae* strains were diverse and associated with the presence of acquired resistance genes and/or amino acid substitutions in antibiotic target proteins (Fig. 2). The ribosomal multilocus sequence typing (rMLST) tree showed high genomic diversity, with no dominant clones or clear division by year, sex, or source. Resistance phenotypes and their genetic determinants mapped to multiple, distantly related lineages, consistent with extensive horizontal acquisition of MGEs rather than clonal expansion of a single MDR lineage.

β-Lactam resistance was common, particularly to ampicillin, detected in 89.4% of MDR strains (Table 1). Resistance to amoxicillin/clavulanic acid was detected in 49.4% and cephalosporin resistance was also frequent, with 55.3% of strains resistant to both cefuroxime and cefotaxime, and 50.6% to ceftriaxone. All strains were susceptible to meropenem. The most prevalent resistance determinant was *bla*_TEM-1_, while *bla*_CTX-M-15_ and *bla*_ROB-1_ were detected only in a small number of isolates. Multiple substitutions were also identified in PBP3.

Resistance to macrolides was observed in 57.6% of MDR strains. The most common genetic determinants identified were the efflux pump genes *mef*(E) and *msr*(D), detected together in 42 resistant strains. No known resistance mechanism was identified in five resistant strains (HUB-HP15538, HUB-HP17005, HUB-HP17315, HUB-HP17520, and HUB-HP17586). Additionally, two resistant strains (HUB-HP16407 and HUB-HP13736) only had the *acrB* R321S mutation, a substitution previously associated with macrolide efflux. However, this same mutation was also found in two susceptible strains (HUB-HP15203 and HUB-HP15205), questioning its potential role in resistance. No previously described point mutations were detected in ribosomal targets (23S rRNA or ribosomal proteins L4/L22) in any of these strains.

In the remaining antibiotic families studied, the identified resistance determinants explained the phenotypic profiles. Ciprofloxacin resistance was associated with substitutions in the quinolone resistance-determining regions of GyrA and ParC, and strains with ParC substitutions exhibited higher ciprofloxacin minimum inhibitory concentrations (MICs). No plasmid-mediated quinolone resistance genes were detected. Chloramphenicol resistance was associated with *catS* or *catA2*. Cotrimoxazole resistance was linked to *sul2* in some isolates and, more frequently, to alterations in *folA* and *folP*. Finally, tetracycline resistance was highly prevalent and mainly associated with *tet*(B)/(C)/(R) or *tet*(M), with 11 strains carrying both determinants.

### ICEs, Tn, and resistance plasmids

**Fig 3:**
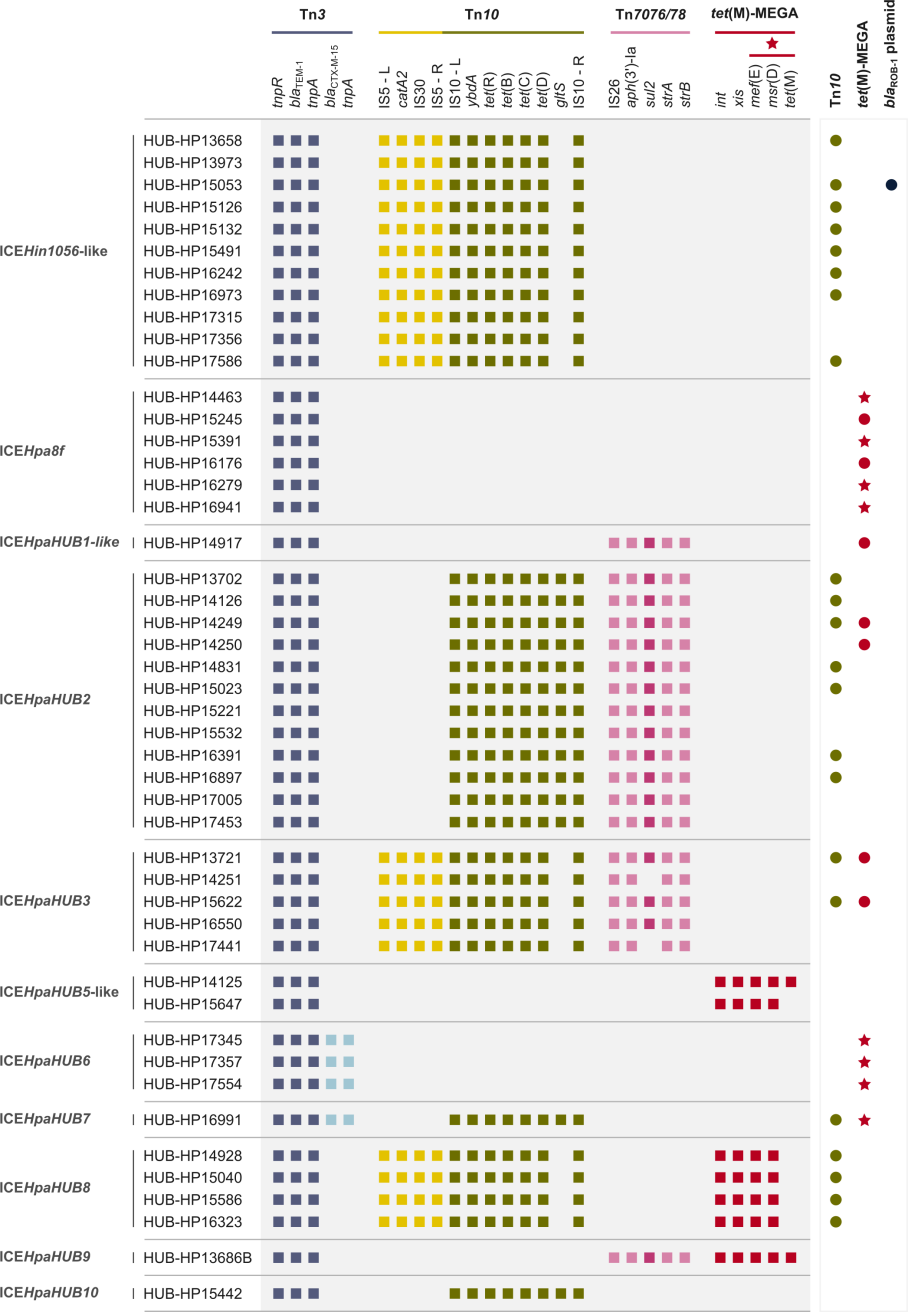
Distribution of integrative and conjugative elements (ICEs) in *Haemophilus parainfluenzae*. The matrix shows resistance-associated genes detected in the study strains. Rows are strains (grouped by ICE type); columns are genes organized by module [Tn*3*, Tn*10*, Tn*7076*-Tn*7078*, and *tet*(M)-MEGA]. Colored squares within the dark-gray panel indicate genes detected within the ICE of each strain. At the right, markers summarize elements detected outside ICEs (chromosomal insertions): colored circles indicate the presence of the corresponding resistance module or plasmid *bla*_ROB_; the star indicates a *tet*(M)-MEGA module that includes *mef*(E), *msr*(D), and *tet*(M) but lacks the excision/integration genes *xis* and *int*.

**Fig 4:**
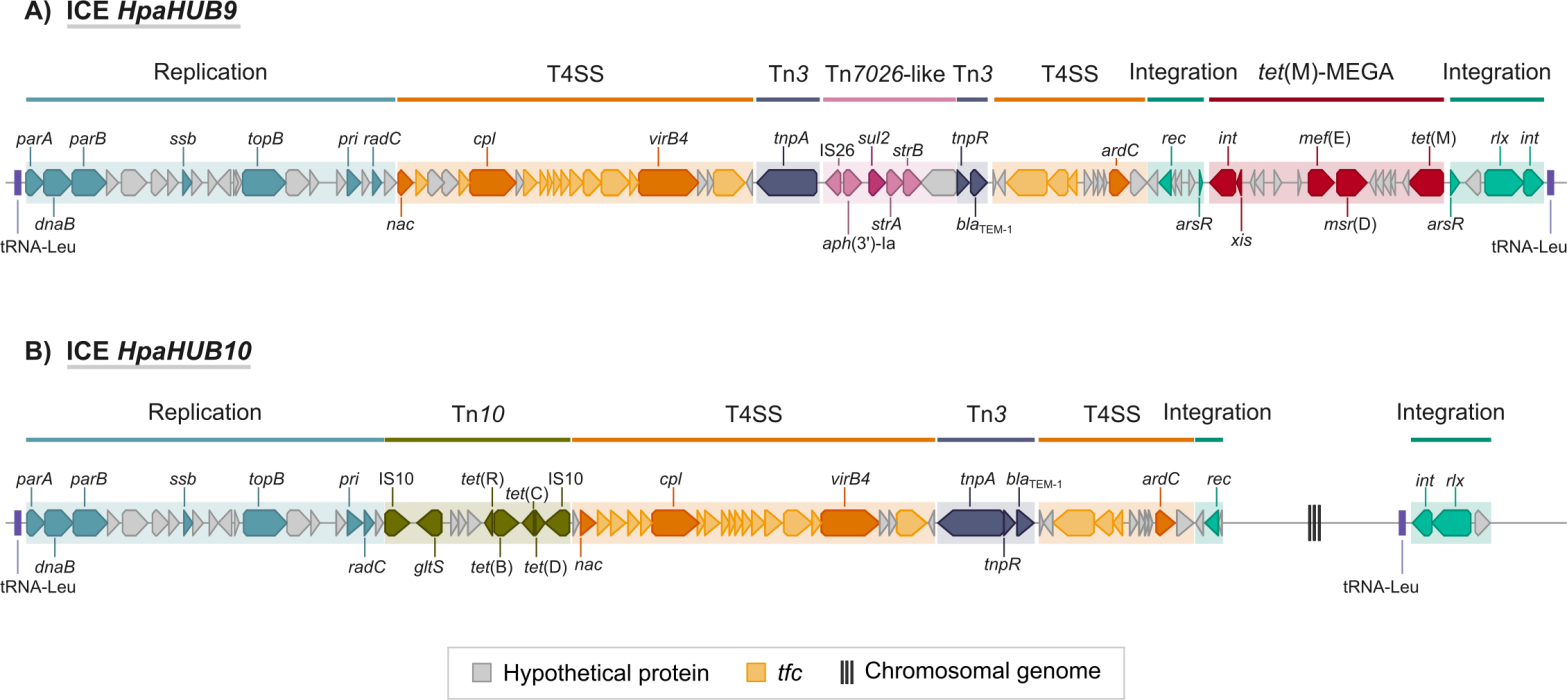
Genetic organization of the new integrative and conjugative elements (ICEs) ICE*HpaHUB9* and ICE*HpaHUB10*. Both elements are integrated at a tRNA-Leu site and display the modular organization typical of ICEs. The replication/maintenance module contains *parA* (plasmid partition protein A), *dnaB* (replicative DNA helicase), *parB* (plasmid partition protein B), *ssb* (single-stranded DNA-binding protein), *topB* (DNA topoisomerase), *pri* (primase), and *radC* (DNA-repair protein). The type IV secretion system is divided into two clusters containing *nac* (nitrogen-assimilation protein), *cpl* (coupling protein), *virB4* (T4SS ATPase), *ardC* (antirestriction proteins), and *tfc* genes (“type four conjugation” genes). The integration/excision module contains *rec* (serine recombinase), *asrR* (transcriptional repressor), *rlx* (relaxase), and *int* (integrase). Hypothetical proteins are shown in gray. (**A**) ICE*HpaHUB9* carries a Tn*3* transposon containing *tnpA* (transposase), *tnpR* (resolvase), and *blaTEM-1* (β-lactamase TEM type I); a Tn6026-like element harboring IS26 (transposase), *aph*(3′)-Ia (aminoglycoside phosphotransferase), *sul2* (sulfonamide-resistant dihydropteroate synthase enzyme), *strA/aph*(3″)-Ib (aminoglycoside phosphotransferase), and *strB/aph*(6)-Id (aminoglycoside phosphotransferase), and a *tet*(M)-MEGA element carrying *int* (tyrosine-type recombinase/integrase), *xis* (excisionase), *mef*(E), *msr*(D) (macrolide efflux genes), and *tet*(M) (tetracycline ribosome protection protein). (**B**) ICE*HpaHUB10* carries a Tn*3* transposon and a Tn*10* transposon containing IS10 transposases, *glts* (sodium-glutamate symporter), and *tet*(B)/(C)/(D)/(R) (efflux system involved in tetracycline extrusion). In the strain where ICE*Hpa*HUB10 was detected (HUB-HP15442), the integration module was disrupted by 740,111 bp of the core genome.

Among MDR genomes, a diverse set of ICEs was identified in 47/85 (52.9%) strains, including ICE*Hin1056*-like, ICE*Hpa8f,* and multiple ICE*HpaHUB* variants (Fig. 3; Fig. S2). All detected ICEs presented a Tn*3* module encoding *bla*_TEM-1_. Notably, two previously unreported ICEs were identified and designated as ICE*HpaHUB9* and ICE*HpaHUB10* (Fig. 4). These elements contained multiple resistance modules, including *bla*_TEM-1_ and tetracycline resistance determinants. Transposons and the *tet*(M)-MEGA element (Fig. S3) were also detected outside ICEs as independent chromosomal insertions in strains with and without ICEs.

**Fig 5:**
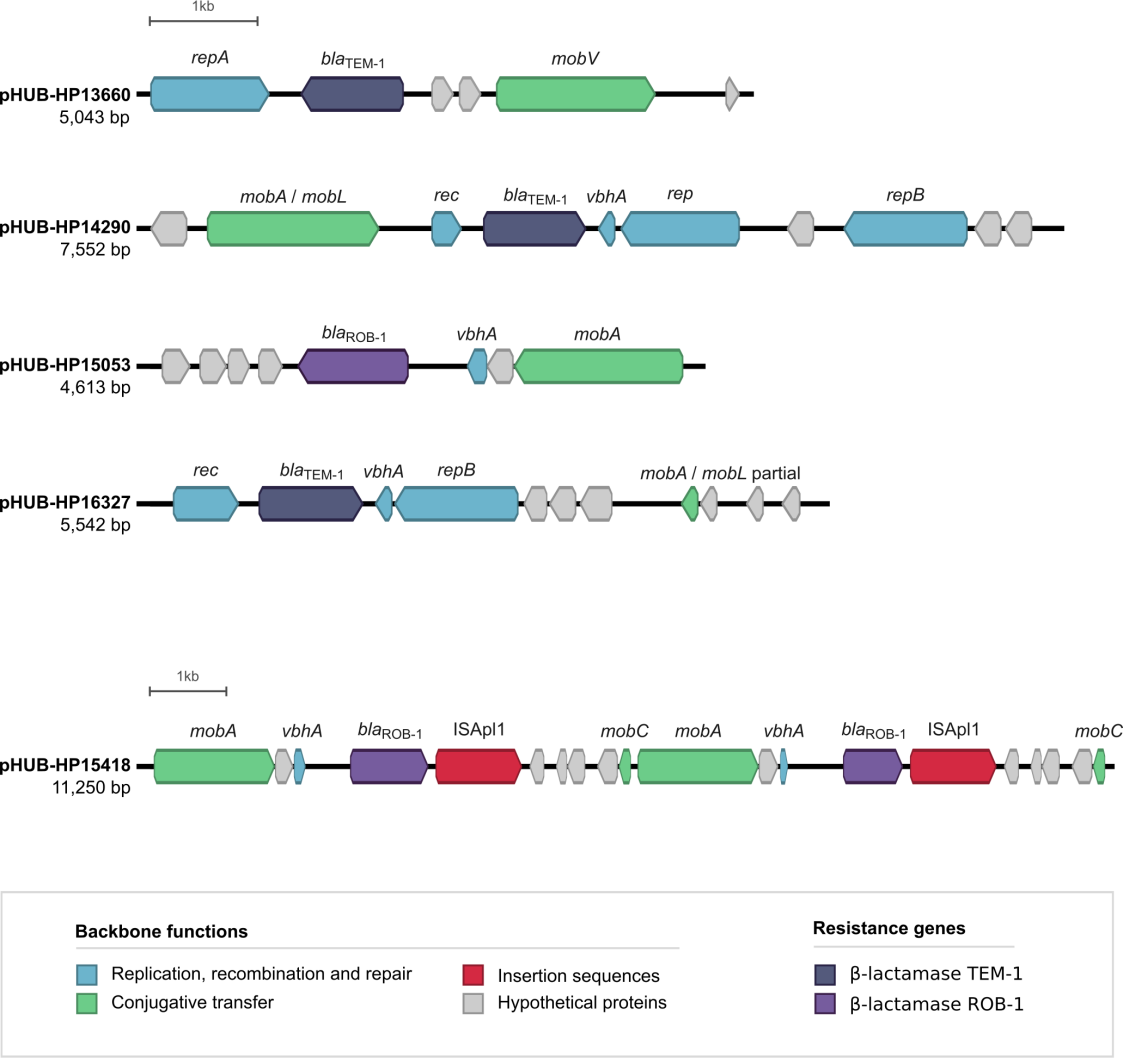
Genetic organization of β-lactamase plasmids identified in multidrug-resistant *Haemophilus parainfluenzae* isolates. Linear maps of plasmids pHUB-HP13660, pHUB-HP14290, pHUB-HP15053, pHUB-HP16327, and pHUB-HP15418 showing annotated open reading frames in the direction of transcription. Plasmid sizes (bp) are indicated next to each map. Backbone functions include replication and maintenance genes (*repA*, *rep*, *repB*, *repC*, *rec*, and *vbha*), conjugative transfer genes (*mobA*, *mobL*, *mobV*, and *mobC*), and hypothetical proteins. Resistance genes corresponding to bla_TEM-1_ or bla_ROB-1_ and the insertion sequence ISApl1 are highlighted in distinct colors.

Small β-lactamase-containing plasmids were also identified (Fig. 5; Table S3) in some urogenital strains. The most frequent plasmid (*n* = 6) was a 5,043 bp *bla*_TEM-1_ plasmid with 38.8% GC, sharing 99.9% nucleotide identity with *H. influenzae* plasmid pJ612 (JQ783056). Strain HUB-HP14290 harbored a 7,552 bp plasmid (38.2% GC) that was 96.6% identical to *N. gonorrhoeae* pBla plasmid from strain WHO_V_2024 (CP145022); unlike the gonococcal pBla, which encodes TEM-135, the HUB-HP14290 plasmid carried *bla*_TEM-1_. HUB-HP16327 carried a 5,542 bp *bla*_TEM-1_ plasmid (40.3% GC) showing 81.1% identity to the gonococcal plasmid PJD4 (Asian-TEM135-MIC64, MK973079). Finally, two strains contained ROB-type β-lactamase plasmids: in HUB-HP15053, we identified a 4,615 bp plasmid (41.5% GC) 99.9% identical to *Glaesserella parasuis* pB1000 (KP164832), and HUB-HP15418 carried an 11,250 bp plasmid (41.3% GC), sharing 99.8% identity with the 5,685 bp APP7_A plasmid from *Actinobacillus pleuropneumoniae* (CP001094). In pHUB-HP15418, the plasmid backbone contained a tandem structure, consistent with the incorporation of two related plasmid segments into a single 11.2 kb replicon. None of the *bla*_CTX-M-15_ genes detected in this study were plasmid-borne.

In strain HUB-HP15203, we also detected a 4,488 bp chromosomal resistance region (positions 1,884,348–1,888,835) inserted between the genes *iivL* (acetolactate synthase 3 large subunit) and *fabB* (β-ketoacyl-ACP synthase I). This region consisted of a cluster including *tnpR*, *bla*_TEM-1_, *repC*, *sul2*, and *strA* (Fig. S4).

Overall, β-lactamase determinants in the MDR collection were located either within ICE-associated Tn*3* modules or on small plasmids, and no additional plasmids carrying antimicrobial-resistance genes were detected (Fig. S5).

### Prevalence of capsulated *H. parainfluenzae*

A total of 35 (6.6%) *H. parainfluenzae* isolates carrying a capsular operon were identified. The majority were collected from urogenital samples (*n* = 32, 91.4%), while only three (8.6%) were obtained from respiratory samples. Overall, 26 of the 35 capsulated isolates were MDR.

Most capsulated strains (*n* = 32) harbored the *H. parainfluenzae* HPAR_type1 capsular operon, which includes four serotype-specific genes in region II (Fig. S6). The operon structure was highly conserved, with the exception of two strains (HUB-HP14126 and HUB-HP16897), which carried an insertion of an IS4 family transposase (ISVa5) within the *pcsB* gene (Fig. S7). In HUB-HP14126, the insertion occurred at nucleotide position 6117 and generated an alternative open reading frame (ORF) of 1,377 bp. In HUB-HP16897, the transposase was inserted at position 6193, resulting in an alternative ORF of 2,862 bp.

A single urogenital strain (HUB-HP17268) had a different capsular operon, the recently described *H. parainfluenzae* HPAR-type4 (PV567605) (28), related to *H. sputorum* HSPU_type1 (NZ_AFNK01000000). This operon had a total length of 11,885 bp, contained three serotype-specific genes in region II, and belonged to group 2 in the *Haemophilus* spp. classification of capsular operons (13).

Among the respiratory strains, two of them (HUB-HP13629 and HUB-HP16013) presented a serotype f-like capsular operon, similar (>96%) to the one found in the strain PT-11747 from Portugal. In this work, we propose to designate this operon as *H. parainfluenzae* HPAR_type3 to establish a uniform nomenclature. This operon had a total length of 15,490 bp and includes five predicted ORFs in region II. Comparative analysis showed that it shares the highest sequence similarity with an operon from *H. haemolyticus* (NZ_SDPB01000001.1, >83%), which we designated as HHAE_type1. Both operons belong to group 2 based on their genetic structure. The remaining respiratory strain (HUB-HP16973) carried the HPAR_type1 operon.

**Fig 6:**
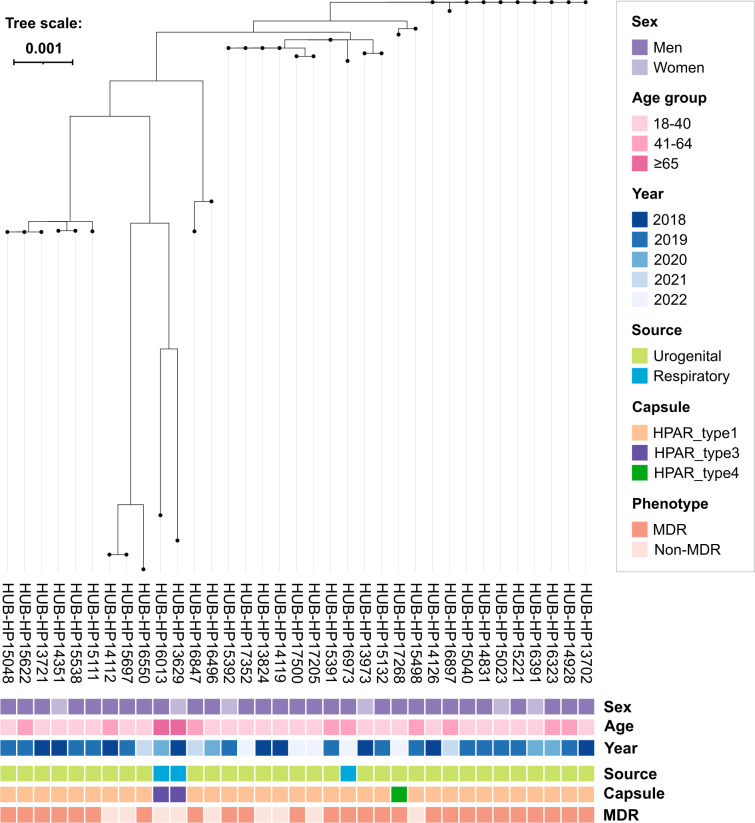
rMLST phylogeny of capsulated *Haemophilus parainfluenzae* strains (2018–2022). The dendrogram was inferred from pairwise allelic distances across the 53 ribosomal loci (rMLST, PubMLST scheme). Tip labels indicate strain IDs. Colored strips beneath the tree summarize, from top to bottom, sex (men/women), age group (18–40, 41–64, and ≥65), year (2018–2022), source (urogenital or respiratory), capsule operon (HPAR_type1, HPAR_type3, and HPAR_type4), and resistance phenotype (MDR or non-MDR).

Strains carrying a capsular operon did not form a single lineage but were distributed across several independent clades (Fig. 6). HPAR_type1 was the most frequent operon and appeared in multiple branches, largely among urogenital strains. The serotype f-like operon (HPAR_type3) was detected in two strains, forming a small respiratory cluster. HPAR_type4 was detected in a single urogenital strain positioned within the overall diversity of capsulated lineages. MDR and non-MDR capsulated isolates co-occurred across clades, with no clear partition by year or sex.

## DISCUSSION

This 5-year follow-up study consolidates and extends prior observations from our center, showing that *H. parainfluenzae* is a frequent opportunistic pathogen in urogenital niches, and multidrug resistance continues to expand (1, 13). Compared with 2013–2017, the epidemiological pattern in 2018–2022 was broadly similar, with most isolates recovered from respiratory or urogenital samples, a clear source-dependent age distribution, and a persistent male predominance consistent with the burden of urethritis and other STI presentations (1, 9, 11).

During this period, MDR remained largely concentrated in the urogenital niche. The overall MDR rate was 16.1% and markedly higher in prevalence among urogenital isolates than among respiratory or other samples. Compared with 2013–2017, the overall pattern appears stable, with a modest increase in MDR in the recent period (1, 13). Notably, in 65.3% of symptomatic urogenital MDR episodes, *H. parainfluenzae* was the only STI-related organism detected in culture, compared with 25.0% symptomatic cases in the early period (1). Similar trends have been reported in other studies, with *H. parainfluenzae* frequently recovered as the unique isolate in urethritis and other urogenital infections (9–11, 29). Co-existence with classical STI pathogens in the urogenital mucosa may promote horizontal gene transfer, given the species’ natural competence and the burden of MGEs documented in this study and previously (13–15, 18, 19). Taken together, STI-like clinical presentations, frequent co-infections, and the availability of mobile resistance elements support the clinical relevance of *H. parainfluenzae* as an STI-associated pathogen and reinforce the need for its diagnostic consideration.

The highest resistance rates were observed for ciprofloxacin, tetracycline, ampicillin, and cotrimoxazole, in line with the general profile previously described in our center (1, 13, 21). In contrast, chloramphenicol resistance decreased compared with 2013–2017 (1). Temporal evaluation of macrolide resistance was not possible, as routine phenotypic testing was not performed during the previous period. Although direct comparisons across studies are limited by differences in sampling strategies and antibiotic panels, similar resistance patterns have been reported, particularly highlighting increasing resistance to β-lactams, fluoroquinolones, and tetracyclines among urogenital *H. parainfluenzae* isolates (10, 12, 24, 29–31).

In recent years, cephalosporin resistance increased in both respiratory and urogenital isolates. Similar concerns have been reported in other settings, where reduced cephalosporin susceptibility has been linked to PBP-associated substitutions (32). In this context, the detection of *bla*_CTX-M-15_ ESBL (22) in four urogenital isolates represents a secondary, less frequent resistance mechanism that, together with reports from other centers (23, 24, 33), supports the emergence of ESBL dissemination. Although CTX-M producers remain uncommon, their localization within transferable MGEs is concerning because it provides a plausible route for dissemination in the urogenital reservoir. Continued surveillance is therefore recommended to monitor further spread.

Importantly, urogenital isolates in our setting are predominantly recovered from individuals at high risk of sexually transmitted infections, who are frequently exposed to empirical antibiotic regimens, particularly third-generation cephalosporins (ceftriaxone) and tetracyclines (doxycycline). This sustained antibiotic pressure may contribute to the selection and maintenance of resistant strains and raises concerns regarding the potential impact on empirical STI treatment efficacy (34, 35).

Macrolide resistance in our collection was predominantly associated with the *mef(*E)-*msr*(D) efflux system. However, a subset of resistant isolates lacked any known acquired resistance determinants or canonical mutations in ribosomal targets. In these strains, only the *acrB* R321S substitution was identified in some cases, although its presence in susceptible isolates questions its role in resistance. These findings suggest that additional or yet uncharacterized mechanisms may contribute to macrolide resistance in *H. parainfluenzae* (19, 36). Further genomic and functional studies will be required to clarify the underlying mechanisms in these isolates.

ICEs were identified in over half of MDR genomes, mirroring the prevalence observed in 2013–2017 (13). While the percentage remained stable, the current period was characterized by greater structural diversity. In addition to ICE*Hin1056*-like, ICE*Hpa8f*-like, and multiple ICE*HpaHUB* structures previously described in *H. parainfluenzae* (13), we detected six new variants (ICE*HpaHUB5-10*). Among these, ICE*HpaHUB9* and ICE*HpaHUB10* are new structures described in this study, whereas the others were reported previously by our group (19, 22). This diversity supports ongoing evolution of resistance through modular rearrangement and exchange. Resistance modules such as Tn*10* and *tet*(M)-MEGA were detected both within ICEs and as independent chromosomal insertions, reinforcing the mosaic resistance architectures characteristic of the genus (13–15, 18, 19). Notably, the *tet*(M)-MEGA module identified in ICE*HpaHUB9* showed a genetic organization consistent with previously described MEGA-associated elements such as Tn*2009*, although with an inverted orientation. The presence of integrase (*int*) and excisionase (*xis*) genes suggests that this module may behave as an integrative and mobilizable element (37). However, functional studies would be required to confirm its mobility and precise classification. All these findings align with the well-documented natural competence of *Haemophilus* spp. and explain the broad phylogenetic dispersion of resistance profiles despite the absence of a single dominant MDR clone (15, 38–40).

Additionally, small β-lactamase plasmids were identified in a subset of urogenital MDR genomes. The most common was a ~5 kb pJ612-like *bla*_TEM-1_ plasmid previously reported in *H. influenzae* (GenBank JQ783056), consistent with the molecular organization of small *bla*_TEM-1_ plasmids described in *Haemophilus* spp. (17, 41). Less frequently, we detected gonococcal p_*bla*-like replicons and ROB β-lactamase plasmids related to pB1000, previously documented across Pasteurellaceae and in clinical *H. influenzae* isolates (42, 43). One of the ROB β-lactamase plasmids (pHUB-HP15418) harbored two identical tandem modules, suggesting possible ISApI1-mediated duplication of the *bla*_ROB-1_ resistance cassette. These findings indicate that β-lactamase plasmids co-circulate and diversify within urogenital bacterial communities. Given the niche overlap and frequent co-colonization of *H. parainfluenzae* and *N. gonorrhoeae*, small resistance plasmids represent concerning reservoirs for interspecies dissemination of β-lactamase genes (42, 44). Notably, all detected resistance plasmids were small, in contrast with prior studies in *Haemophilus* reporting larger plasmids lacking resistance determinants (28, 45). This pattern is consistent with the fitness costs of plasmid-borne resistance that limit long-term maintenance unless compensated by adaptive evolution (46).

Capsular operons were identified in 6.6% of the isolates, predominantly among urogenital samples and, for the first time in our center, in three respiratory isolates. This represents an increase from 2013 to 2017, when capsules were present in less than 2% of the strains, all from urogenital samples (1, 13). In addition, two operons not detected in the earlier period were identified (HPAR_type3 and HPAR_type4), indicating expanded ecological range and operon diversity. Although capsule carriage in *H. parainfluenzae* has been mainly associated with urogenital isolates, capsulated strains have also been reported in respiratory colonizing contexts, including a nasopharyngeal isolate from children in Portugal (27). In this context, HPAR_type1 remained the most prevalent operon in our collection, consistent with previous observations suggesting that capsule carriage is enriched in urogenital isolates and may contribute to niche adaptation (25). Notably, capsular loci related to those described in our collection have also been reported in other settings, including France and Portugal, confirming that capsulated *H. parainfluenzae* is not geographically restricted (23, 27). As the number of capsular operons proliferates across studies, standardized nomenclature is essential for unambiguous cross-study comparison. We therefore propose a unified nomenclature whereby capsular operons are referred to as HPAR_typeX in order of detection (e.g., HPAR_type1-4), with region II (serotype-specific) genes annotated with an operon-specific suffix (e.g., *pscA1* and *pscB1* for HPAR_type1), while conserved regions I and III retain the conventional *bexABCD* and *hcsAB* designations. Under this system, the previously termed *H. parainfluenzae* serotype f-like operon (27) is standardized as HPAR_type3, facilitating consistent reporting as additional operons are described. Finally, although capsular loci were identified at the genomic level, the functional expression of capsule could not be assessed, and the impact of transposase insertions within some HPAR_type1 operons remains unknown.

Phylogenetically, rMLST revealed a highly heterogeneous population structure among MDR isolates, with resistance determinants detected across distantly related lineages and only a minor respiratory-enriched cluster. This broad phylogenetic dispersion, together with the modular structure of ICEs and transposons, supports that recurrent horizontal acquisition predominates, rather than the expansion of a single successful clone, consistent with previous comparative genomics in this species (13, 17, 19). Similarly, capsule loci occurred across multiple lineages rather than a single clonal background, reflecting their diversity (13, 26, 27). In addition, capsulated isolates were identified among both MDR and non-MDR lineages, suggesting that capsule carriage does not restrict the acquisition of antimicrobial resistance. Future studies are needed to evaluate the impact of distinct capsular operons on transmissibility, virulence, and niche adaptation, especially within the urogenital tract.

This study has several limitations. As a single-center retrospective analysis based on routine diagnostic data rather than systematic case definitions, certain pathologies may have been underestimated. Specifically, incomplete clinical information for urogenital cases limited the estimation of the overall prevalence of *H. parainfluenzae* in urethritis. In addition, information on prior antibiotic exposure was not consistently available, precluding a robust comparison between MDR and non-MDR cases and limiting the assessment of antibiotic exposure as a potential risk factor. rMLST captures population structure at conserved loci; higher-resolution core-genome approaches could refine fine-scale transmission signals. Furthermore, our genomic analysis targeted only MDR and capsulated isolates, so non-sequenced isolates may harbor undetected ICEs or other resistance elements. Finally, comparisons between periods should be interpreted with caution due to potential differences in case identification and sampling. The decline in respiratory isolates may reflect COVID-19 disruptions and physician changes, rather than a true epidemiological shift. Prospective, multicenter studies with comprehensive WGS across all isolate categories will be important to address these limitations.

This 5-year follow-up confirms *H. parainfluenzae* as being predominantly associated with urogenital infections and documents MDR spread. Rather than arising from clonal expansion, resistance emerges by chromosomal mutations and recurrent horizontal acquisition mediated by ICEs, transposons, and small β-lactamase plasmids, highlighting an evolving genetic landscape with implications for transmission and persistence. Our study documents the dispersion of resistance and capsule loci across lineages, identifies small plasmids as relevant resistance vehicles, and introduces a pragmatic HPAR_type nomenclature to harmonize capsule identification. These findings argue for strengthened surveillance of *H. parainfluenzae* in STI clinics to anticipate the spread of resistance and prevent interspecies exchange among STI-associated pathogens. Future multicenter surveillance studies will be essential to better define the epidemiology, resistance trends, and dissemination of relevant clones and mobile genetic elements in this species.

## MATERIALS AND METHODS

### Study design and strain classification

This was a retrospective laboratory-based study that included all MDR *H. parainfluenzae* strains collected between 2018 and 2022 at Hospital Universitari de Bellvitge (HUB), a tertiary care hospital located in Southern Barcelona (Spain). Non-MDR strains carrying putative capsular operons were also included for further genomic characterization. Clinical samples were processed by standard procedures, and individual colonies were isolated and identified as *H. parainfluenzae* by MALDI-TOF mass spectrometry (Bruker Daltonics, Germany). Species identification was further supported by WGS data. Samples were classified into three categories based on their origin: (i) respiratory, (ii) urogenital, and (iii) other clinical samples. Isolates obtained from the same patient within 30 days were considered the same episode, and only the first strain was included in the analysis.

Clinical charts of patients with MDR *H. parainfluenzae* were reviewed, including demographic and clinical variables according to sample origin. Three age groups were established: young adults (18–40 years), middle-aged adults (41–64 years), and elderly adults (65 years or more).

A schematic overview of the study design and analytical workflow is provided in Fig. S1.

### Antibiotic susceptibility testing

Antimicrobial susceptibility was routinely tested by disk diffusion using Mueller-Hinton fastidious agar (BioMérieux, France; 5% horse blood and 20 μg/mL of β-NAD). MDR was defined as resistance to three or more antibiotic families, and XDR was defined as resistance to five or more antibiotic families. MICs were analyzed for all MDR strains by microdilution using STRHAE2 Sensititre commercial panels (Thermo Fisher Scientific, USA). The antibiotics tested included β-lactams, fluoroquinolones, macrolides, chloramphenicol, cotrimoxazole, and tetracycline. All procedures for susceptibility studies were performed in accordance with the European Committee on Antimicrobial Susceptibility Testing criteria for *H. influenzae* (v15.0, 2025; www.eucast.org/clinical_breakpoints).

### Capsular *bexA* gene screening

PCR screening for the capsular *bexA* gene was performed on all *H. parainfluenzae* strains collected between 2018 and 2022. The following primers were used: HUB12445-FW (5′-CCGCTTGGATTAGTGGCGTTG-3′) and HUB12445-RV (5′-ACTGGGACTGTGTGAAACCA-3′). Chromosomal DNA from strain HUB1114 (HPAR_type1, ERS4413375), a capsulated strain carrying the *bexA* gene, was used as the positive control.

### Whole-genome sequencing

All MDR *H. parainfluenzae* strains, as well as non-MDR strains that tested positive for the *bexA* gene, were selected for WGS. DNA was extracted with the QIAamp DNA Mini Kit (QIAGEN) and quantified using Qubit 4 Fluorometer (Thermo Fisher Scientific). Libraries were prepared with the Nextera XT DNA library preparation kit (Illumina, USA), with subsequent short-read sequencing on a MiSeq platform (2 × 300 bp). FastQ sequences were assembled with the INNUca (v4.2.0) pipeline (https://github.com/B-UMMI/INNUca).

After bioinformatics analysis of the sequences, MDR strains encoding acquired antibiotic resistance genes and capsulated strains were selected for long-read sequencing. The Native Barcoding Expansion (EXP-NBD196) and the Ligation Sequencing Kit (SQK-LSK109) were used for library preparation, followed by sequencing on FLO-MIN106D flow cells (R9.4.1) for a 48 h run (Oxford Nanopore Technologies, UK). A hybrid assembly of Illumina and Oxford Nanopore Technologies was conducted using the Unicycler pipeline (https://github.com/rrwick/Unicycler). Short and long reads were deposited at the European Nucleotide Archive (PRJEB107088). Final genome assemblies were uploaded to the *Haemophilus influenzae* PubMLST database (see Data set S1).

### Phylogenetic analysis

Population structure was analyzed in two independent data sets: (i) MDR strains and (ii) strains carrying a putative capsular operon. For each data set, rMLST was performed on the PubMLST platform (https://pubmlst.org/) using the *Haemophilus* rMLST scheme (53 ribosomal protein loci). Allele calling followed the platform’s default pipeline.

Phylogenetic analysis was performed using rMLST on the PubMLST platform. Trees were visualized using iTOL (v6, https://itol.embl.de/), with metadata overlays indicating sample source, year, MDR/XDR status, and capsule-operon type. Final layouts were refined in Inkscape.

### Characterization of resistance determinants

Acquired resistance mechanisms were screened using Abricate (v0.8.0, https://github.com/tseemann/abricate) with the ResFinder (v3.2) database (47). Resistance-associated point mutations were analyzed with Geneious R9 (v9.1.7; Biomatters, USA) using *H. parainfluenzae* T3T1 (NC_015964) closed genome as the reference. ICEs were also studied with Geneious R9 using the *ICEHpa8f* (AM884335) and ICE*Hin1056* (AJ627386) sequences as references.

### Capsular operon determination

HiCap (https://github.com/scwatts/hicap) was used to identify the capsular locus *in silico* (48). In addition, the structure of the operon was analyzed with Geneious R9, using as references the sequence of the *H. parainfluenzae* HPAR-type1 capsular operon (from the HUB12445 [MH644108)], *H. parainfluenzae* HPAR-type2 (from the HUB10748 [MT185932]), and *H. parainfluenzae* serotype f-like (from the PT11747 [JAMLEO010000002:224525-240017]). The sequences of the capsular operons of *H. influenzae* serotype a (CP017811), serotype b (NC_016809), serotype c (HQ651151), serotype d (HM770877), serotype e (FM882247), serotype f (CP005967:675016-687441), *H. sputorum* HSPU_type1 (NZ_AFNK01000000), *H. sputorum* HSPU_type2 (QEPN01000003), and *H. haemolyticus* (NZ_SDPB01000001.1) were also analyzed using Geneious R9 to compare all capsular operons within the genus. The ORFs were predicted, and the percentage of identity between genes from different capsular operons was calculated as the identical number of bases divided by the length of the longest gene.

### Statistical analysis

GraphPad software (https://www.graphpad.com/) was used to perform the two-tailed Fisher’s exact test to compare categorical variables when appropriate.

## SUPPLEMENTAL MATERIAL

Data set S1 spectrum.01039-26-s0001.xlsx Clinical, microbiological, and antimicrobial resistance data of all multidrug-resistant and capsulated *Haemophilus parainfluenzae* isolates included in the study.

Supplemental material spectrum.01039-26-s0002.pdf Fig. S1 to S7; Tables S1 to S3.

ASM does not own the copyrights to Supplemental Material that may be linked to, or accessed through, an article. The authors have granted ASM a non-exclusive, world-wide license to publish the Supplemental Material files. Please contact the corresponding author directly for reuse.
