# Supplementary material for: Epidemiology and antimicrobial resistance trends in Haemophilus parainfluenzae in a tertiary care hospital (2018–2022)
Source: Microbiol Spectr. 2026 Jul 15;14(8):e01039-26. doi: 10.1128/spectrum.01039-26 (PMC13435796; doi:10.1128/spectrum.01039-26)
Supplement: Supplementary file 1 [file spectrum.01039-26-s0002.pdf]

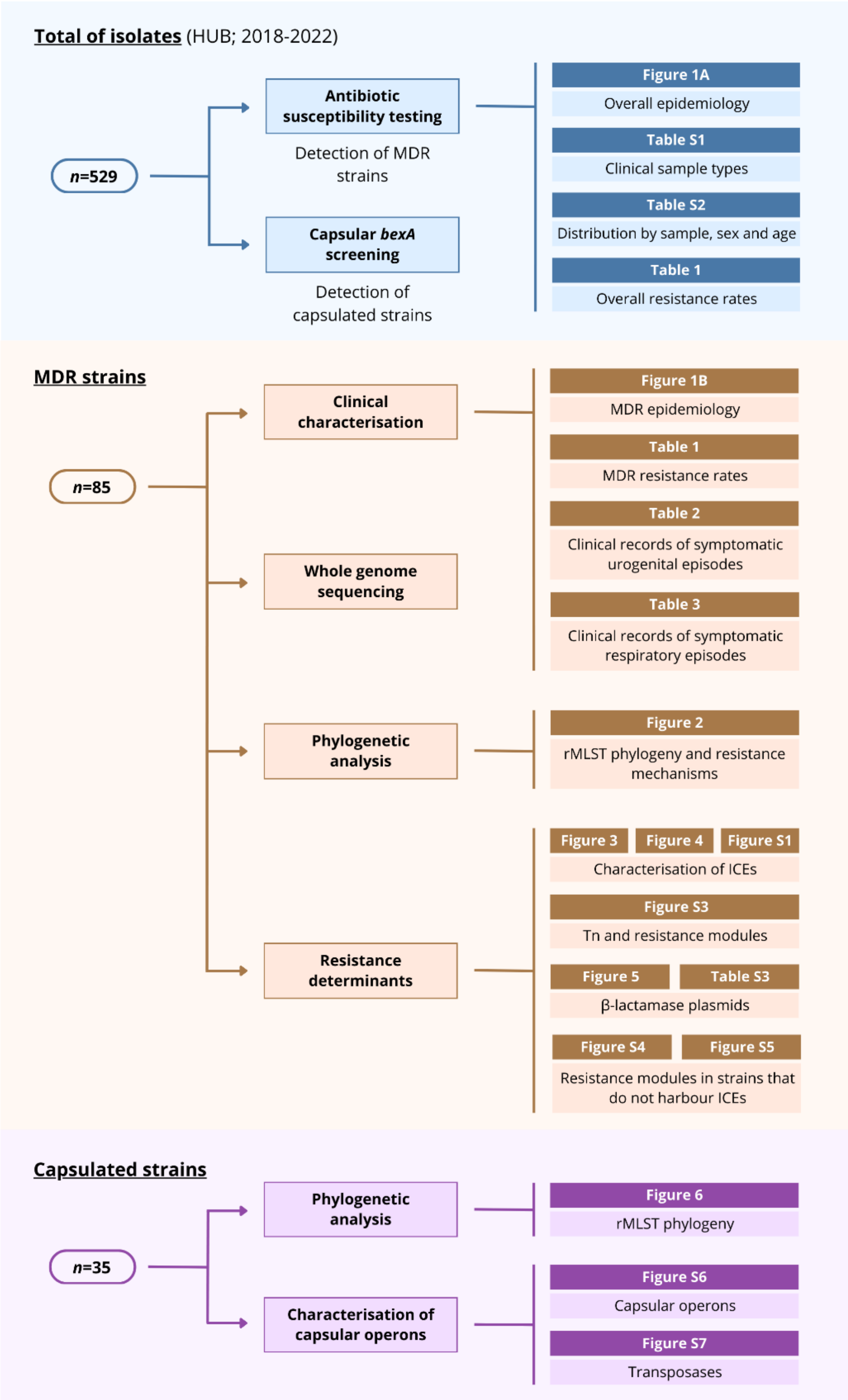

Figure S1. Study design and schematic workflow of *Haemophilus parainfluenzae* isolates collected between 2018 and 2022 at Hospital Universitari de Bellvitge (HUB). All isolates underwent antimicrobial susceptibility testing and *bexA* PCR screening to identify multidrug-resistant (MDR) and capsulated strains. MDR isolates (*n* = 85) were further analysed by clinical review, whole-genome sequencing, phylogenetic analysis and characterisation of resistance determinants. Capsulated isolates (*n* = 35) were analysed separately by phylogenetic reconstruction and capsular operon characterisation. Corresponding main and supplementary figures and tables are indicated.

Table S1. Detailed distribution of clinical sample types of *Haemophilus parainfluenzae* isolates (2018-2022).

Sample group	Sample type	*n* (%) [a]
Respiratory (*n* = 258)	Sputum	159 (61.6)
	Bronchial aspirate	77 (29.8)
	Tracheal aspirate	16 (6.2)
	Bronchoalveolar lavage	2 (0.8)
	Pharyngeal swab	2 (0.8)
	Bronchial biopsy	1 (0.4)
	Pleural fluid	1 (0.4)
Urogenital (*n* = 223)	Urethral swab	110 (49.3)
	Vaginal swab	67 (30.1)
	Balanopreputial swab	18 (8.1)
	Genital ulcer	13 (5.8)
	Endocervical swab	5 (2.2)
	Rectal swab	5 (2.2)
	Semen	2 (0.9)
	Bartholin gland swab	2 (0.9)
	Perianal swab	1 (0.4)
Other (*n* = 48)	Bile	11 (22.9)
	Surgical wound	9 (18.7)
	Abscess pus	8 (16.7)
	Blood	3 (6.2)
	Peritoneal fluid	3 (6.2)
	Conjunctival pus	2 (4.2)
	Pancreatic pus	2 (4.2)
	Appendicular pus	1 (2.1)
	Articular biopsy	1 (2.1)
	Subcutaneous tissue biopsy	1 (2.1)
	Synovial biopsy	1 (2.1)
	Appendicular pus	1 (2.1)
	Fistula pus	1 (2.1)
	Traumatic wound	1 (2.1)
	Cubital ulcer	1 (2.1)
	Foot ulcer	1 (2.1)
	Urine from nephrostomy	1 (2.1)

[a] Percentages are calculated within each sample group.

Table S2. Distribution of *Haemophilus parainfluenzae* isolates by sample origin, sex and age group (2018-2022).

A. Sex distribution by sample origin.

Sample group	Age group (years)	Male, *n* (%)	Total, *n* (%) [a]
Respiratory (*n* = 258)	18-40	11 (91.7)	12 (4.7)
	41-64	67 (67.0)	100 (38.8)
	≥65	101 (69.2)	146 (56.6)
Urogenital (*n* = 223)	18-40	108 (69.7)	155 (69.5)
	41-64	38 (66.7)	57 (25.6)
	≥65	4 (36.4)	11 (4.9)
Other (*n* = 48)	18-40	2 (50.0)	4 (8.3)
	41-64	20 (76.9)	26 (54.2)
	≥65	9 (50.0)	18 (37.5)

[a] Percentages are calculated within each sample group.

B. Median age by sample origin.

Sample group	Median age [IQR]
Respiratory (*n* = 258)	66.5 [59.0-72.0]
Urogenital (*n* = 223)	32.0 [25.0-45.0]
Other (*n* = 48)	61.0 [50.5-73.5]

Abbreviations: IQR: interquartile range.

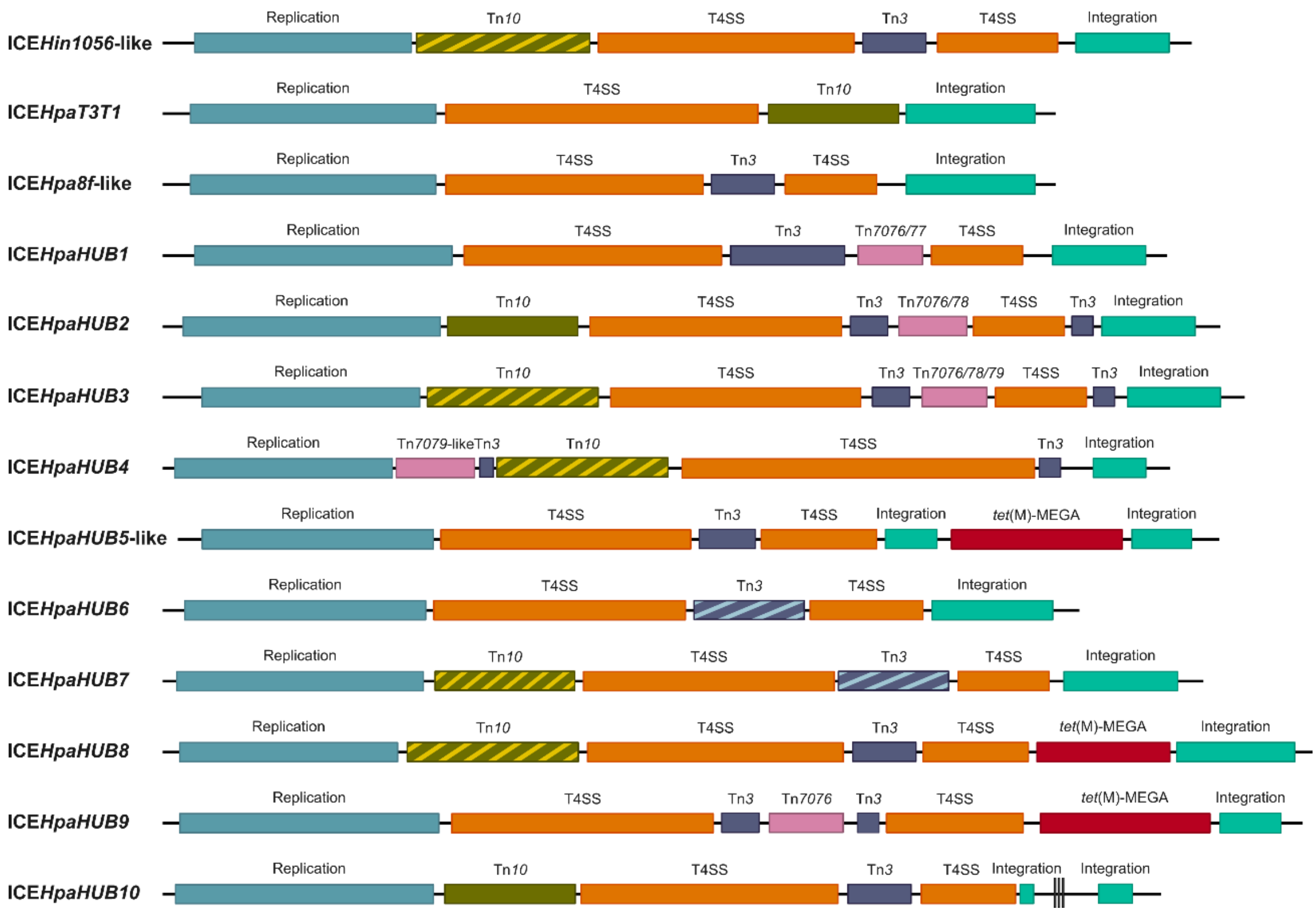

Figure S2. Schematic representation of integrative and conjugative elements (ICEs) identified in MDR *Haemophilus parainfluenzae* at Hospital Universitari de Bellvitge (2013-2022). Blocks are coloured by functional modules including replication/maintenance, type IV secretion system (T4SS), resistance elements, and integration/excision. Resistance modules include Tn*3*, Tn*10*, Tn*7076-79*, and the *tet*(M)-MEGA element; their gene content and organisation are detailed in Figure S2.

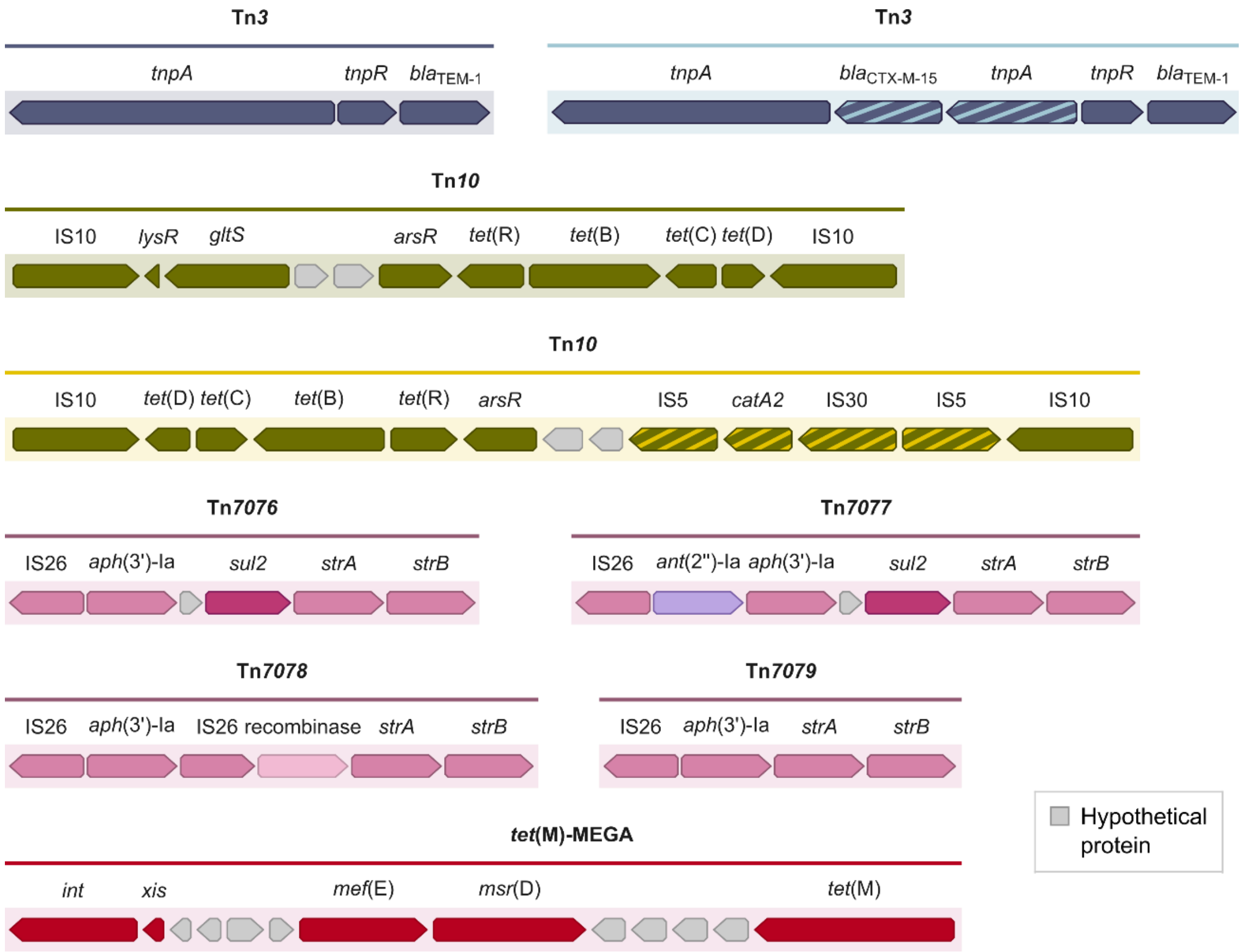

Figure S3. Representative transposons and resistance modules detected in MDR *Haemophilus parainfluenzae*. Linear maps show the gene organisation of Tn*3*, Tn*10*, Tn*7076-79*, and the *tet*(M)-MEGA element. Arrows indicate open reading frames and transcriptional orientation; and grey boxes denote hypothetical proteins. Tn*3* is represented with two configurations: (i) a classical Tn*3* carrying *bla*$_{TEM-1}$, and a composite Tn3 harbouring *bla*$_{CTX-M-15}$ together with *bla*$_{TEM-1}$, each with *tnpA*/*tnpR* transposition genes. Tn*10* is shown in two variants: (i) one encoding the tetracycline efflux/regulatory *tet*(B)/(C)/(D)/(R) and an (ii) expanded variant that also carries *catA2*. Tn*7076*-Tn*7079* modules encode aminoglycoside/sulphonamide/streptomycin determinants (*aph*(3')-Ia or *ant*(2")-Ia, *sul2*, *strA*/*strB*). The *tet(*M)-MEGA element comprises *mef*(E), *msr*(D) and *tet*(M).

Table S3. BLASTn alignments showing the closest matches for plasmid sequences identified in multidrug resistant *Haemophilus parainfluenzae* genomes.

Study collection			BLASTn					
**Strain**	**Plasmid**	**Length (bp)**	**Predicted plasmid**	**Length (bp)**	**Species**	**Identity (%)**	**Coverage (%)**	**Accesion Sequence Reference**
HUB-HP13660	pHUB-HP13660	5043	pJ612	5048	HINF	99.85	100.00	JQ783056.1
HUB-HP14290	pHUB-HP14290	7552	pBla WHO_V_2 024	7427	NGON	96.56	98.00	CP145022.1
HUB-HP15053	pHUB-HP15053	4615	pB1000	4613	GPAR	99.86	100.00	KP164832.1
HUB-HP15194	pHUB-HP13660	5043	pJ612	5048	HINF	99.87	100.00	JQ783056.1
HUB-HP15418	pHUB-HP15418	11250	plasmid APP7_A	5685	APLE	99.81	100.00	CP001094.1
HUB-HP15538	pHUB-HP13660	5043	pJ612	5048	HINF	99.87	100.00	JQ783056.1
HUB-HP16283	pHUB-HP13660	5043	pJ612	5048	HINF	99.87	100.00	JQ783056.1
HUB-HP16327	pHUB-HP16327	5542	pJD4	5542	NGON	99.94	100.00	MK973079.1
HUB-HP16847	pHUB-HP13660	5043	pJ612	5048	HINF	99.85	100.00	JQ783056.1
HUB-HP17454	pHUB-HP13660	5043	pJ612	5048	HINF	99.87	100.00	JQ783056.1

Abbreviations: HINF: *Haemophilus influenzae*; NGON: *Neisseria gonorrhoeae*; GPAR: *Glaesserella parasuis*; APLE: *Actinobacillus pleuropneumoniae*.

BLASTn searches were performed against the NCBI nt database. For each plasmid, the closest match is shown together with the corresponding reference plasmid, species, nucleotide identity, coverage, and accession number.

Plasmids designated pHUB-HP13660 were detected in multiple strains and showed >99.90% pairwise nucleotide identity to each other and ~99.85% identity to plasmid pJ612.

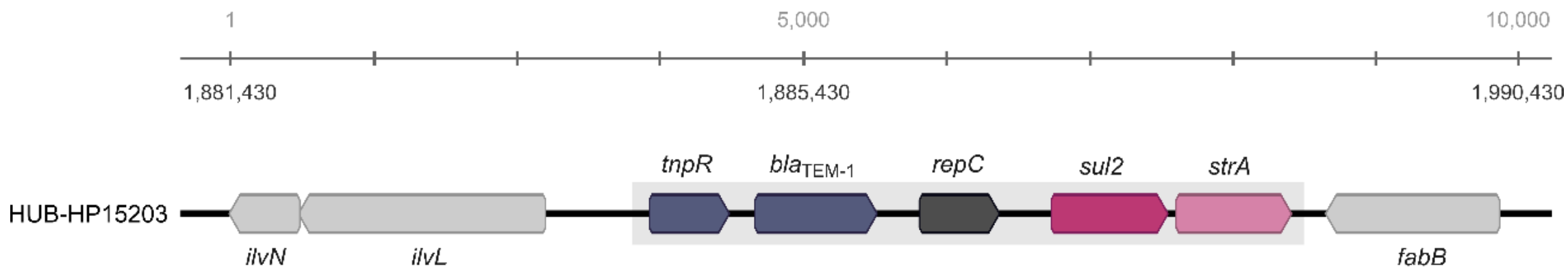

Figure S4. Genetic organisation of the chromosomal resistance region found in strain HUB-HP15203. Schematic representation of a 4,488 bp resistance region inserted in the chromosome of strain HUB-HP15203 between the genes *ilvL* (acetolactate synthase 3 large subunit) and *fabB* (β-ketoacyl-ACP synthase I). The shaded segment comprises a *tnpR*-*bla*$_{\text{TEM-1}}$-*repC*-*sul2*-*strA* gene cluster, where *tnpR* encodes a resolvase, *bla*$_{\text{TEM-1}}$ a TEM-1 β-lactamase, *repC* a plasmid replication protein, *sul2* a sulfonamide resistant dihydropteroate synthase and *strA* an aminoglycoside phosphotransferase. Flanking genes are shown in grey.

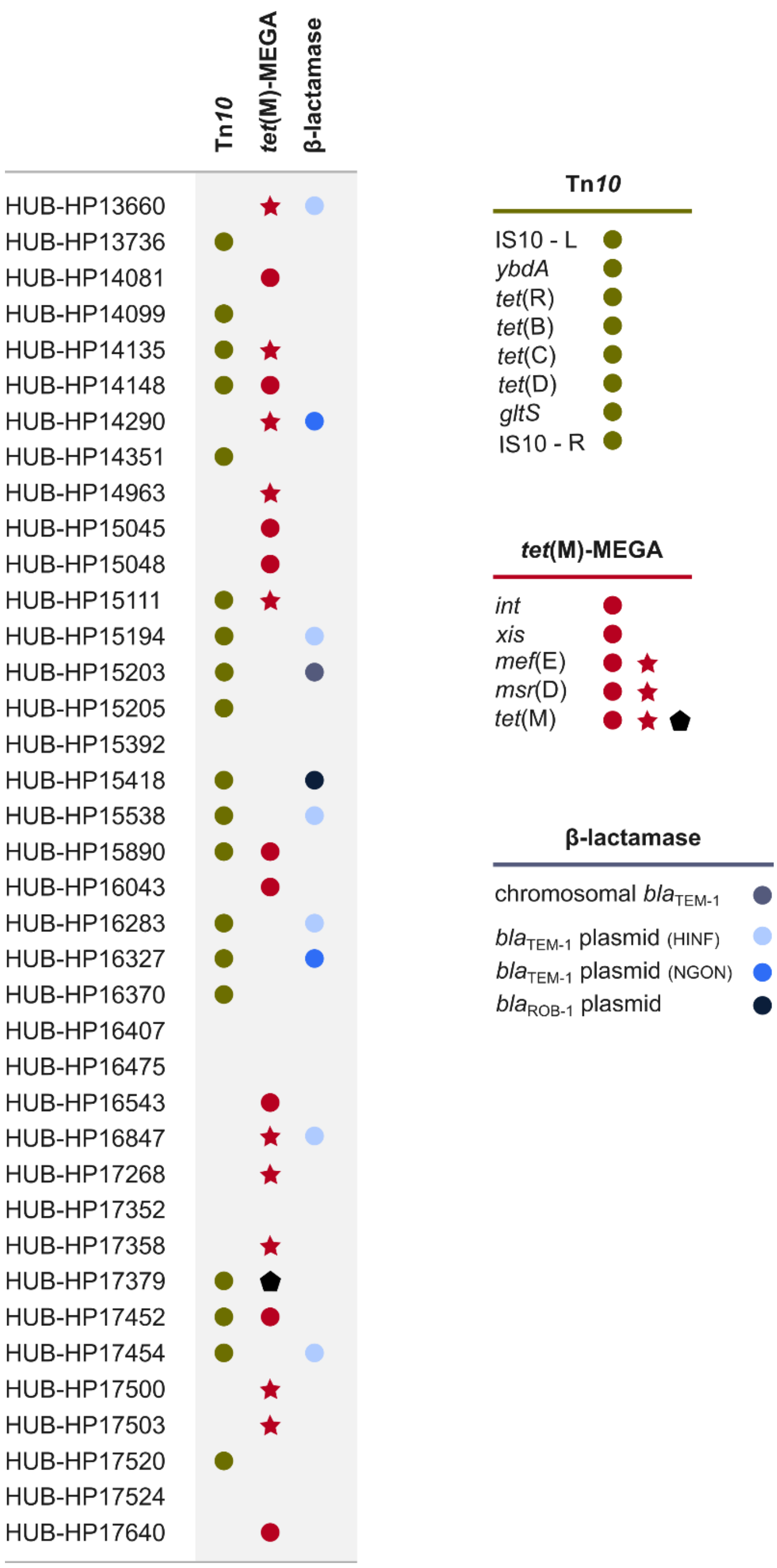

Figure S5. Distribution of Tn*10*, *tet*(M)-MEGA and β-lactamase vehicles across MDR *Haemophilus parainfluenzae* genomes that do not harbour integrative and conjugative elements. Rows list strain IDs; columns indicate the presence/absence of the resistance determinants. All detected Tn*10* elements corresponded to the variant with *tet*(B)/(C)/(D)/(R) and without *catA2*; *tet*(M)-MEGA was observed in multiple configurations, including the complete module (*int/xis*, *mef(E)*, *msr(D)* and *tet(M)*) and partial derivatives. β-lactamase vehicles included chromosomal *bla*TEM-1, *bla*TEM-1 plasmids related to *H. influenzae* and *N. gonorrhoeae*, and a *bla*ROB-1 plasmid related to *Actinobacillus pleuropneumoniae*.

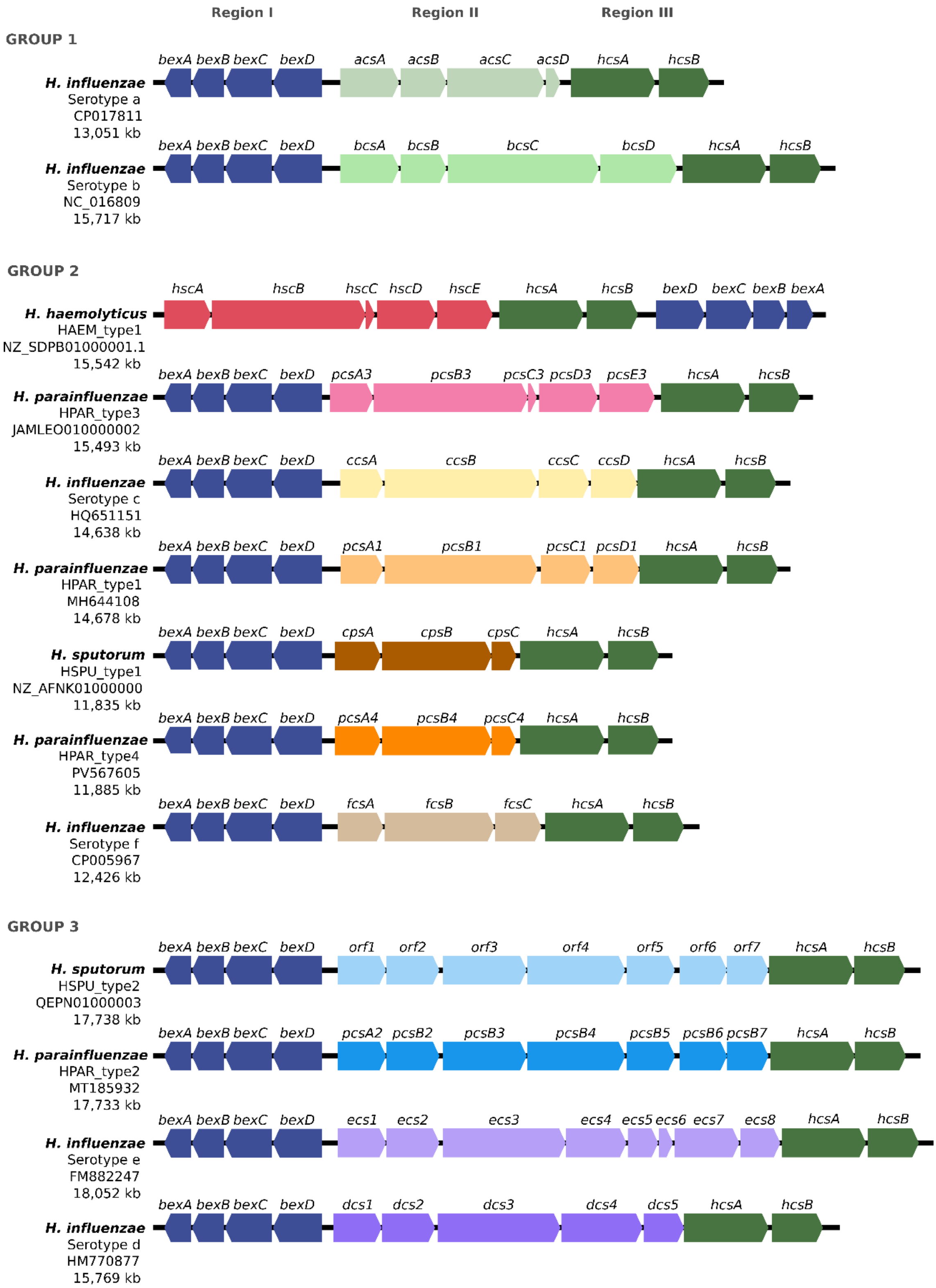

Figure S6. Genetic classification of the *Haemophilus* spp. capsular operons. The operons have been categorized into three groups based on similarities in their genetic structure. Group 1 comprises *H. influenzae* serotypes a and b, Group 2 includes *H. haemolyticus* HAEM_type1, *H. parainfluenzae* HPAR_type1, HPAR_type3, and HPAR_type4, along with *H. sputorum* HSPU_type1 and *H. influenzae* serotypes c and f, while Group 3 comprises *H. sputorum* HSPU_type2, *H. parainfluenzae* HPAR_type2, and *H. influenzae* serotypes d and e. The three main regions are depicted in different colours.

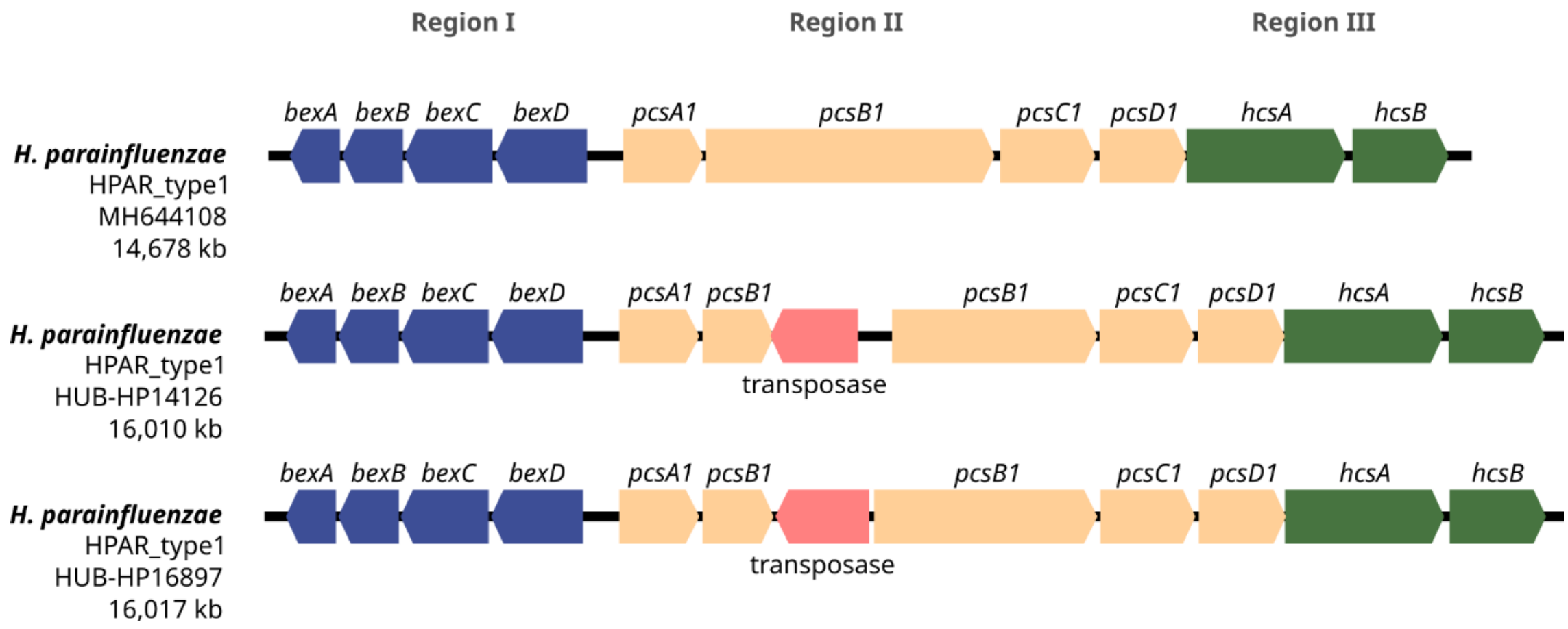

Figure S7. Transposase insertion in *H. parainfluenzae* HPAR_type1 capsular operons. HUB-HP14126 and HUB-HP16897 had an IS4-family transposase (ISVa5) is inserted within *pcsB* in Region II, disrupting the gene. Arrows indicate ORFs and transcriptional orientation; colours distinguish the three *cap*-locus regions.
